# The effect of acute branched-chain amino acids ingestion on rate of force development in different time intervals: a controlled crossover study

**DOI:** 10.3389/fnut.2024.1463202

**Published:** 2025-01-08

**Authors:** Xi-Nuan Zhang, Long-Ji Li, Yan-Hao Tu, Li-Feng Zhang, Hua-Yu Shang, Meng Liu, Ming-Da Li

**Affiliations:** ^1^School of Sports Training, Chengdu Sport University, Chengdu, China; ^2^Strength and Conditioning Training Center, School of Physical Education, Chengdu Sport University, Chengdu, China; ^3^School of Sports Medicine and Health, Chengdu Sport University, Chengdu, China; ^4^Chongqing Institute of Sports Science, Chongqing, China

**Keywords:** branched-chain amino acids, BCAAs, sports nutrition, rate of force development, controlled cross-over study

## Abstract

**Background:**

Branched-chain amino acids (BCAAs) are widely used as sports nutrition supplements. However, their impact on the rate of force development (RFD), an indicator of explosive muscle strength, has not yet been validated. This study aimed to assess the impact of BCAA supplementation on the RFD in college basketball players during simulated games.

**Methods:**

This study employed a randomized, controlled crossover, double-blind design. Participants received either BCAAs (0.17 g/kg combined with 0.17 g/kg isocaloric glucose) or a placebo (0.34 g/kg isocaloric glucose) orally 30 min before beginning the exercise protocol. The RFD was quantified using the isometric mid-thigh pull (IMTP) test. Additional outcome measures, including strength and jump tests, agility and sprinting tests, and physiological responses, were also assessed. A two-way repeated measures ANOVA was employed to evaluate the impact of supplements (BCAAs and placebo) on RFD and other related outcome measures.

**Results:**

Analysis of the 50 ms RFD demonstrated significant main effects of BCAA supplementation (*p* = 0.003). The BCAAs group consistently exhibited higher levels of 50 ms RFD compared to the placebo group across rounds 1 to 4. For example, in round 1, the 50 ms RFD was 3702.3 ± 1223.2 N/S in the BCAAs group versus 2931.3 ± 888.8 N/S in the placebo group (*p* = 0.045). Although no significant between-group differences were observed for the 100, 150, 200, and 250 ms RFD measurements, the BCAAs group consistently showed superior values across all time points. The results of other outcome indicators also suggested that supplementation with BCAAs was indeed effective.

**Conclusion:**

The results indicate that BCAA supplementation can enhance RFD in basketball players, particularly at the 50 ms RFD. Our research design provides reliable insights into the effects of BCAAs on athletic performance. Further studies of similar design with larger sample sizes are necessary to confirm and extend these findings.

**Clinical trial registration:**

Chinese Clinical Trial Registry, ChiCTR2400091314 (https://www.chictr.org.cn).

## 1 Introduction

As we all know, movements such as sprinting, changing direction, and jumping require athletes to generate and transmit large forces in very short periods, usually between 30 and 250 ms (1, 2). Therefore, rate of force development (RFD) has been used to directly reflect explosive strength (3) and is widely used in team sports (4–7). Based on different time intervals (such as 0–50, 0–100, 0–150, 0–200, and 0 − 250 ms), RFD can be categorized into different types (5, 8–11). Generally, early RFD (≤100 ms) and late RFD (>100 ms) reflect different aspects of muscle explosive performance, thus suggesting distinct underlying mechanisms (12, 13).

Early RFD is often primarily attributed to neural mechanisms (12). Some studies suggested that early RFD is particularly sensitive to neuromuscular fatigue (12, 14). Neuromuscular fatigue is generally considered to result from a combination of peripheral and central fatigue (15), with central fatigue being directly related to neuromuscular fatigue. Central fatigue is defined as the loss of force or strength proximal to the neuromuscular junction (16), leading to a decline in muscle force production capacity (17). The primary mechanisms of central fatigue may include reflex effects from muscle afferents, stimulation of type III and IV nerves, changes in the excitability of neurons within the motor cortex, the release of cytokines, and alterations in serotonin synthesis (16). During exercise, serotonin-mediated central fatigue involves the continuous release of serotonin, which enhances the synaptic effects of serotonergic neurons and subsequently reduces neural drive and motor unit recruitment (18). The ratio of branch chain amino acids (BCAAs) to tryptophan in the blood is an important factor influencing serotonin release (19). Although late RFD is closely associated with isometric maximal voluntary contraction force (MVF) and is more influenced by structural variables such as muscle cross-sectional area and architectural features (13), early RFD also affects the values of late RFD. Therefore, BCAAs might have an influence on late RFD as well.

BCAAs, consisting of leucine, isoleucine and valine, have been widely used as sports nutrition supplements. As mentioned above, the role of BCAAs in central fatigue has been extensively studied as a way to validate their role in exercise performance. Several reviews have shown interactions between BCAAs, tryptophan and serotonin (20–22). However, the evidence regarding the direct effects of BCAAs on various aspects of exercise performance remains inconsistent (23, 24). For example, recent research shows that acute BCAA supplementation enhances alactic and lactic anaerobic performance but has no effect on peak power, sprinting, or aerobic capacity (24). To the best of our knowledge, the impact of BCAAs on RFD—a critical parameter in strength and conditioning science—has been rarely studied. Furthermore, there is a lack of rigorously designed, high-quality studies in this area.

To fill this knowledge gap and provide a more detailed understanding, the present study aimed to investigate the effects of BCAA supplementation on RFD at different time intervals using a randomized controlled crossover design. This design was chosen because it allows for direct comparisons within subjects, minimizing the confounding effects of inter-individual variability. We hypothesized that supplementation with BCAAs would regulate RFD at different time intervals during exercise, particularly in the early RFD.

## 2 Materials and methods

### 2.1 Study design

This study employed a randomized, controlled crossover, double-blind design. Sixteen participants were assigned in a 1:1 ratio to two groups (the BCAAs group and the placebo group) consisting of computer-generated random codes by independent researchers. BCAAs and placebo (similar taste) were identically packaged and coded with numbers (Supplementary Figure S1A). Participants, researchers, and outcome assessors were blinded to the randomization order and group assignment throughout the study. The crossover design was chosen because it is efficient and requires fewer participants to test the feasibility of the study protocol. In addition, the variability in the effects of the supplement was less than the variability between participants, making a crossover design appropriate.

**Figure 1:**
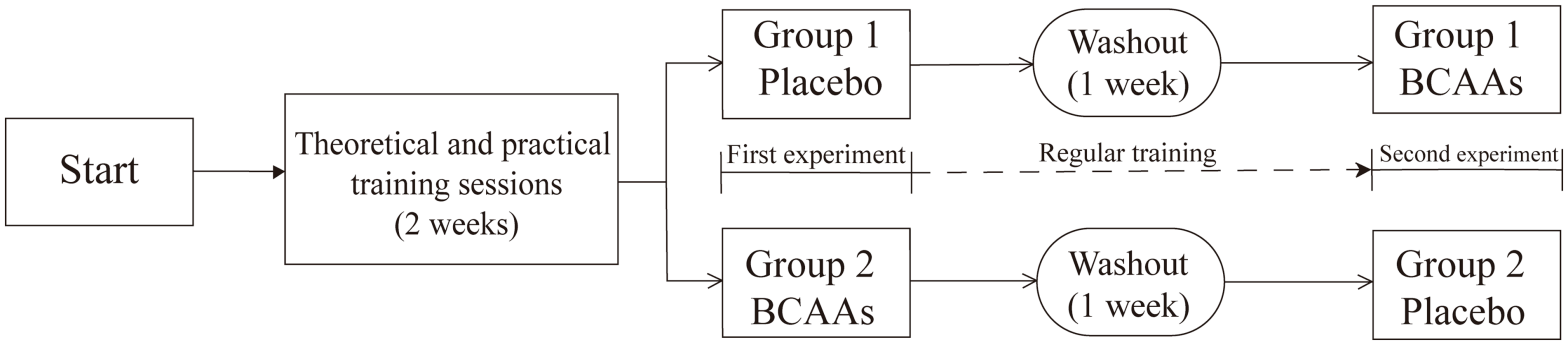
Crossover study design.

The experiment consisted of the theoretical and practical training session and two formal testing phases (Figure 1). To minimize errors due to varying familiarity with the test items, participants received five comprehensive theoretical and practical training sessions over a 2-week period to familiarize them with the standardized protocol. One-week washout period was included between the two testing phases, which was considered sufficient to eliminate any residual effects. Each testing phase comprised five rounds, labeled from round 0 to round 4. Round 0 represented the measurements taken 30 min after BCAAs or placebo, prior to the formal experiment. Rounds 1 to 4 represented the measurements taken after each completed lap.

### 2.2 Participants

Due to practical constraints, including a limited number of female players with both comparable skill levels and sufficient numbers, and the availability of only one male basketball team, we selected 16 male players from the 3×3 basketball team at Chengdu Sport University in Sichuan Province, China, between March and April 2024 (Supplementary Figure S2). Based on the following inclusion criteria: (a) aged 18–25 years; (b) having at least 5 years of basketball training and competitive experience; (c) no medication use; (d) non-smoking; (e) no musculoskeletal injuries in the past 6 months; (f) no major diseases such as cardiovascular, pulmonary and bone diseases. Before the experiment, participants were required to abstain from high-intensity exercise for 1 week. Additionally, they refrained from all sports supplements, including caffeine, creatine, and taurine, for 1 month prior to the experiment. During the 3 days preceding each experiment, dietary intake of each participant was documented and kept as consistent as possible. Throughout the experimental period, the team followed a standard training cycle without scheduled matches. The weekly training regimen remained consistent. Both testing sessions were conducted at the same time of day, following an identical sequence. All participants wore identical athletic attire and basketball shoes, and environmental conditions such as temperature, humidity, and field conditions were kept similar (Supplementary Figures S1B,C). Uniform equipment was used to minimize variability.

Prior to the commencement of the experiment, participants were provided with detailed explanations regarding the potential risks, benefits, scheduling, and procedural details of the study. All participants had the opportunity to review these details and ask questions. The study protocol was approved by the Ethics Committee of Chengdu Sport University [Approval Number: (2024) 57], and written informed consent was obtained from each participant.

### 2.3 Exercise protocol

Each participant in this study completed a structured exercise protocol across five rounds. Each round consisted of three sets of isometric mid-thigh pull (IMTP), four jumping tests, four repetitions of the 505 change of direction (COD) test, and four repetitions of the modified agility T-test. A 30-s recovery period was allowed between rounds, with a 10-s interval between running tests. The entire protocol lasted approximately 18 min of active testing. During the session, participants performed 24 accelerations, 32 decelerations, 64 directional changes, and 20 jumping actions. They received vigorous verbal encouragement and real-time feedback throughout to ensure maximal effort.

Additionally, before each experiment, participants underwent a baseline blood lactate measurement at rest, conducted 40 min prior to the start. After the blood lactate test, participants ingested a specially prepared supplement under the supervision of a professional coach, following a standardized warm-up routine designed to mimic pre-game preparations for 3×3 basketball (25–28). Upon completion of the final round, blood lactate levels and ratings of perceived exertion (RPE) were immediately assessed. After a three-minute rest period, these measurements were repeated.

### 2.4 Supplementation strategy

The strategy involved pre-exercise supplementation where a total volume of 200 mL, containing either BCAAs (0.17 g/kg combined with 0.17 g/kg isocaloric glucose) or a placebo (0.34 g/kg isocaloric glucose), was administered to the participants 30 min before the exercise protocol (29, 30). The BCAAs supplement had a leucine, isoleucine, and valine ratio of 4:1:1, with an absorption time of 30 min, as certified by the supplier (Beijing Competitor Sports Science & Technology Co., Ltd.; Beijing, China). A research team member who was not directly involved in the experiments was responsible for preparing the supplements the day before each experiment.

### 2.5 RFD measurement

The RFD was quantified using the IMTP test, which employed a dual-force platform system that bilaterally captured three-dimensional kinetic data at a sampling frequency of 1,000 Hz (Kistler Instruments Corporation, Amherst, NY, United States). The dual-force platform system was connected to a personal computer running Bioware software (version 4.0.1.2; Kistler Instruments Corporation). The specific measurement procedure was as follows: After a “3, 2, 1, Pull” countdown cue, participants were asked to sustain a maximal effort for a period of 5 s (Supplementary Figure S1D). The test position required the knee and hip angles to remain between 125 and 145 degrees (31). For each maximal effort trial, participants were consistently instructed with the standardized cue, “push your feet into the ground as rapidly and forcefully as possible,” to ensure the attainment of peak RFD. The higher the indicator, the greater the explosive power demonstrated. Finally, to minimize measurement error, each participant was asked to perform three trials.

### 2.6 Other outcomes

Other outcome indicators can be categorized into three aspects: strength and jump tests, agility and sprinting tests, and physiological responses. Strength and jump tests included six indicators: peak force, vertical jump (VJ) fight time, vertical jump (VJ) height, reactive strength index (modify) test (RSImod), peak power, and mean power. Peak force was assessed using the IMTP test, while the other five indicators were evaluated through jumping tests. Higher values of these indicators indicate greater explosive capability. Agility and sprinting tests included two indicators: the 505 change of direction (COD) test and the T test. Shorter completion times for the 505COD test and *T*-test indicated better agility and sprint performance. Physiological responses also included two indicators: blood lactate and the RPE. Blood lactate levels were measured using a portable lactate analyzer (Lactate Scout+; 0017602155; SensLab, GmbH, Germany), the RPE was measured using the Modified Borg Scale (1–10 points). Lower levels or scores on these two indicators indicated better performance.

### 2.7 Statistical analyses

The statistical analyses were conducted using IBM SPSS Statistics 23.0 (Armonk, NY, United States). We used a significance level of *p* < 0.05 to denote statistically significant differences. Post-hoc analysis conducted using G*Power software (version 3.1.9.7, University of Dusseldorf, Dusseldorf, Germany) indicated achieved power ranging from 0.50 to 0.76, with a total sample size of 16 participants (F test, repeated measures, within factors) across various outcome indicators. The study parameters included a large effect size (0.3), a single group, five measurements, and an alpha level of 0.05 (29, 32).

Data were tested for normality using the Shapiro–Wilk test to confirm that all variables were normally distributed. The results were expressed as mean ± standard deviation (SD). Considering the correlation of data from multiple time points and the prerequisites of the model, a two-way repeated measures ANOVA was used to assess the effect of supplements (BCAAs and placebo) and time points (round 0, round 1, round 2, round 3, round 4) on the main outcome indicators (50 ms RFD, 100 ms RFD, 150 ms RFD, 200 ms RFD, and 250 ms RFD). In the preliminary exploration, interaction terms were initially included. However, the majority of these terms were found to be statistically insignificant and unnecessarily complicated the model fit. Therefore, subsequent analyses proceeded using the primary model without interaction effects. Based on these findings, we generated line graphs displaying estimated marginal means. Additionally, we conducted comparisons between groups at each time point, applying Bonferroni corrections to adjust for multiple comparisons. The effect size for the main effects was calculated using partial eta squared (η*_p_*^2^), interpreted as small (0.01–0.06), medium (0.06 < η*_p_*^2^ < 0.14), and large (> 0.14) (33–36). A similar approach was used for the other outcome indicators.

## 3 Results

### 3.1 Participant characteristics

This study used a crossover design and the general characteristics of the participants are detailed in Table 1. The study participants were all male athletes with a mean age of (21.5 ± 1.5) years, height of (1.86 ± 0.1) m, and body mass of (85.8 ± 10.8) kg. Additional comprehensive participant information is provided in Table 1.

**Table 1:** Basic characteristics of participants (*N* = 16).

Variable	Mean ± SD
Gender (male/female)	16/0
Age (years)	21.5 ± 1.5
Height (m)	1.86 ± 0.1
Body mass (kg)	85.8 ± 10.8
BMI (kg/m^2^)	25.1 ± 2.8
Body at percentage (%)	19.6 ± 5.2

### 3.2 RFD in different time intervals

**Figure 2:**
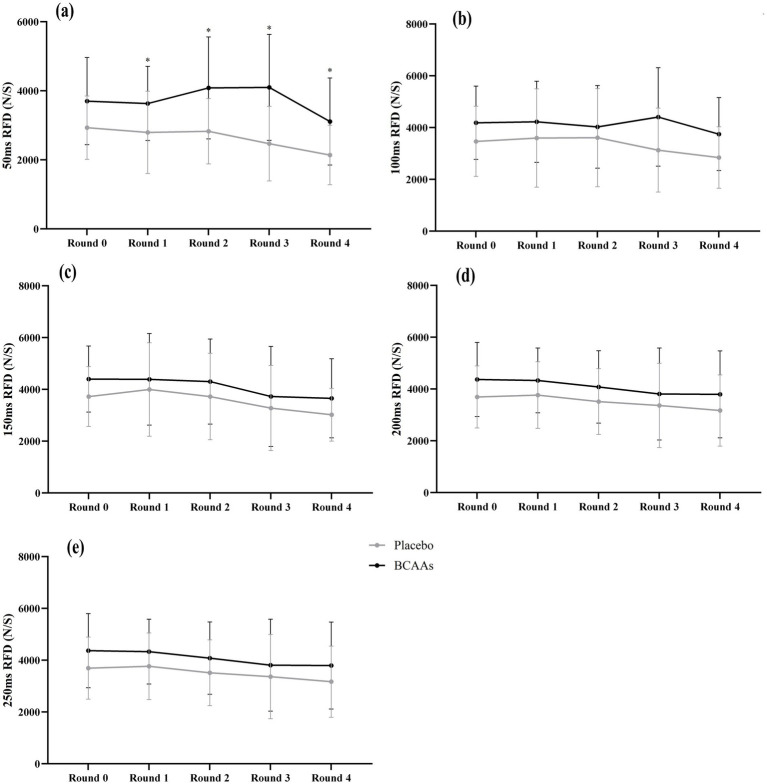
Changes in the rate of force development (RFD) across different time points during the exercise protocol. **(a)** change in 50 ms RFD during the exercise protocol. **(b)** change in 100 ms RFD during the exercise protocol **(c)** change in 150 ms RFD during the exercise protocol **(d)** change in 200 ms RFD during the exercise protocol **(e)** change in 250 ms RFD during the exercise protocol. (*) Indicates statistically significant differences between groups (*p* < 0.05).

Changes in outcome variables (different time intervals) are detailed in Table 2, with corresponding visual representations in Figure 2 for both the BCAAs and placebo conditions. Briefly, the BCAAs group showed a tendency to outperform the placebo group at different time intervals, although most of these differences were not statistically significant, probably due to sample size limitations.

**Table 2:** Changes in outcome variables between placebo and BCAA supplementation conditions in different rounds (mean ± SD).

Parameters	Condition	Round 0	Round 1	Round 2	Round 3	Round 4
50 ms RFD (N/S)	Placebo	2931.3 ± 888.8	2793.9 ± 1154.0	2828.0 ± 915.9	2468.6 ± 1045.6	2141.0 ± 831.9
	BCAAs	3702.3 ± 1223.2	3632.7 ± 1039.9*	4084.5 ± 1429.5*	4099.6 ± 1486.1*	3112.0 ± 1221.0*
100 ms RFD (N/S)	Placebo	3468.5 ± 1311.6	3596.9 ± 1837.9	3611.0 ± 1831.4	3128.9 ± 1569.0	2843.7 ± 1151.8
	BCAAs	4184.4 ± 1370.1	4223.4 ± 1518.2	4024.7 ± 1543.8	4410.6 ± 1841.2	3747.9 ± 1363.6
150 ms RFD (N/S)	Placebo	3723.4 ± 1117.2	3995.2 ± 1746.5	3724.1 ± 1615.5	3279.9 ± 1591.0	3021.9 ± 986.5
	BCAAs	4398.7 ± 1233.3	4389.7 ± 1710.7	4302.9 ± 1590.4	3726.9 ± 1871.3	3653.4 ± 1477.3
200 ms RFD (N/S)	Placebo	3726.7 ± 1142.6	3966.8 ± 1375.9	3688.2 ± 1489.0	3293.7 ± 1539.0	3112.4 ± 1137.1
	BCAAs	4669.4 ± 1352.0	4652.1 ± 1503.9	4562.2 ± 1599.4	3824.3 ± 1741.5	3844.9 ± 1538.5
250 ms RFD (N/S)	Placebo	3691.4 ± 1160.1	3764.5 ± 1244.6	3516.5 ± 1230.2	3364.4 ± 1575.0	3169.9 ± 1331.6
	BCAAs	4368.2 ± 1386.3	4330.4 ± 1210.8	4079.5 ± 1352.6	3807.3 ± 1720.5	3793.1 ± 1625.7

The start of the experiment (round 0) and repeated immediately after each subsequent round (rounds 1–4, respectively); *: <0.05, Bonferroni corrections were applied to account for multiple comparisons.

Analysis of the 50 ms RFD (without an interaction term) showed significant main effects of BCAA supplementation (*p* = 0.003, η*_p_*^2^ = 0.264) and time (*p* = 0.001, η*_p_*^2^ = 0.149) (Figure 2a). The BCAAs group exhibited higher levels of 50 ms RFD compared to the placebo group in rounds 1–4. For example, in round 1, the 50 ms RFD was 3702.3 ± 1223.2 N/S in the BCAAs group, compared to 2931.3 ± 888.8 N/S in the placebo group (*p* = 0.045) (Table 2). For the 100, 150, 200, and 250 ms RFD, no significant between-group effects were observed (Figures 2b–e). However, it was consistently noted that the BCAAs group exhibited superior values compared to the placebo group across all time points.

### 3.3 Other outcomes

Other outcome indicators included three main aspects: strength and jump tests, agility and sprinting tests, and physiological responses (Supplementary Figures S3−S12). No significant differences were observed between the two groups in the measures of strength and jump tests. In certain indicators (such as VJ flight time, VJ height, and RSImod), the BCAAs group consistently exhibited superior values compared to the placebo group across all time points. Agility and sprinting tests included two indicators: the 505 COD and the T test, with shorter completion times indicating better performance. We observed that the BCAAs group had shorter completion times compared to the placebo group, although these differences were not statistically significant. Physiological responses showed that blood lactate levels were significantly lower in the BCAAs group, compared to the placebo group. Additionally, the RPE was also lower in the BCAAs group. These results suggests that BCAA supplementation is effective.

## 4 Discussion

### 4.1 Main findings

To our knowledge, this study is the first rigorously designed, high-quality investigation to examine the effects of BCAA supplementation on RFD in college basketball players during simulated games. We found a significant effect of BCAA supplementation on 50 ms RFD (*p* = 0.003). The BCAAs group consistently demonstrated higher 50 ms RFD values than the placebo group across most of rounds (*p* < 0.05). Although no significant differences were found between groups for the 100, 150, 200, and 250 ms RFD measures, the BCAAs group showed consistently higher values at all-time points. Additional outcome measures further supported the effectiveness of BCAA supplementation.

### 4.2 Comparison with previous work and possible explanations

The current study revealed that BCAA supplementation had a significant effect on the 50 ms RFD, with similar trends observed at different time points. Several observational and experimental studies were consistent with our findings (37–39). In trained individuals undergoing resistance training on a hypocaloric diet, BCAA supplementation helped maintain lean mass and preserved skeletal muscle performance (37). Briefly, the BCAA supplementation group showed increased values in squat and bench press, which were also associated with enhanced explosive muscle strength (37). However, some studies have indicated inconsistent results, suggesting that the effects of BCAAs on muscle performance may be limited or even ineffective (40, 41). For instance, when combined with heavy resistance training over an 8-week period, supplementation with 9 g/day of BCAAs 30 min before and after exercise, did not show any specific benefits on body composition and muscle performance (40). These inconsistent results may be partly attributed to factors such as small sample sizes, variability in outcome measures, and differences in study designs. For instance, previous studies predominantly employed randomized controlled studies, whereas our study utilized a randomized controlled crossover design, further minimizing individual confounding factors and potentially enhancing the reliability of the results. The significant effect of BCAA supplementation on the 50 ms RFD is also biologically plausible. Research suggests that early RFD (≤100 ms) is particularly vulnerable to neuromuscular fatigue (12, 14), which typically results from a blend of peripheral and central fatigue (15). Central fatigue, linked directly to neuromuscular fatigue, includes reflex responses from muscle receptors, activation of type III and IV nerves, changes in neuronal excitability in the motor cortex, release of cytokines, and adjustments in serotonin synthesis (16). During intermittent high-intensity exercise, plasma free fatty acid levels may significantly increase, which in turn can elevate plasma tryptophan concentrations (42). During high-intensity exercise, muscle glycogen serves as the primary substrate, and substantial glycogen depletion within a short period increases the utilization of BCAAs as an energy source (43, 44). In our study, the scenario involved intermittent high-intensity exercise, which likely results in both an increase in the tryptophan concentrations and a reduction in BCAA concentrations. Thus, BCAA supplementation increases the concentration of BCAAs in the blood and lowers the tryptophan/BCAAs ratio, thereby competing with tryptophan for transport across the blood–brain barrier via the same L-system (45). This competition limits tryptophan delivery to the brain, a critical step in serotonin synthesis, and subsequently reduces central fatigue (45).

While no statistically significant differences were observed between groups in the measures of 100, 150, 200, and 250 ms RFD, the BCAAs group consistently exhibited higher values across all time points. These negative results may be attributed in part to sample size limitations. Although we used a randomized controlled crossover design to improve statistical power, the final inclusion of 16 participants was slightly off from the expected 20. Further G*Power analyses indicated that the statistical power ranged from 0.50 to 0.76, which somewhat diminished our ability to detect positive results. The regulatory mechanisms of early RFD (≤100 ms) and late RFD (>100 ms) may differ, providing an alternative explanation. For instance, early rate of force development (RFD) predominantly arises from neural mechanisms (12, 46), whereas late RFD is intricately linked to isometric maximal voluntary contraction force (MVF) and is significantly influenced by structural factors like muscle cross-sectional area and architectural characteristics (13). Overall, future studies with similar designs and larger sample sizes are necessary to validate and extend these findings.

### 4.3 Practical implications

These findings have significant practical implications for basketball athletes and sports practitioners, particularly in the context of optimizing performance. The enhancement of the 50 ms RFD through BCAA supplementation highlights its potential role in improving rapid muscle response and explosive movement capabilities. These attributes are critical in basketball, where agility, quickness, and the ability to generate force rapidly are essential for success during high-pressure game situations, such as sudden changes in direction, jumping, or sprinting. Improved 50 ms RFD may contribute to better on-court performance by enabling athletes to react faster to opponents’ movements, execute plays more effectively, and maintain high-intensity performance throughout games. This suggests that BCAA supplementation could serve as a valuable tool in the training and nutritional strategies of basketball players, offering benefits beyond traditional recovery and muscle repair.

Future research should explore the long-term effects of BCAA supplementation in combination with specific strength and conditioning programs. Understanding how different dosages, timing, and training adaptations influence RFD and other performance metrics could help refine strategies for optimizing athletic output. Additionally, investigating the effects of supplementation across various sports and player positions may provide broader insights into its utility in competitive sports. By integrating these findings into evidence-based practices, athletes and coaches can enhance performance and achieve greater success in high-stakes competition.

### 4.4 Strengths and limitations

The highlight of this study is its focus on explosive power in basketball players during the 0–250 ms period, exploring RFDs over different time intervals and providing comprehensive evidence for explosive performance research. In addition, the study used a rigorous randomized, crossover, placebo-controlled, and double-blind design to ensure the accuracy and reliability of the results. However, there are also several limitations. First, this study was conducted at a single center and included exclusively young male basketball players. Therefore, the generalizability of our findings to other regions and populations, such as females and children, may be limited. Second, the final sample size of this study was limited for practical feasibility reasons, which may have reduced the ability to detect significant results. Fortunately, most of the trends observed after BCAA supplementation were generally consistent with expectations. Finally, we were unable to collect certain biological samples, such as blood and urine, or conduct comprehensive physical examinations. This limited our ability to gather additional data to support our findings. Future studies with larger sample sizes, including a broader range of sports disciplines, both male and female participants, and multiple outcome measures, are needed to confirm these findings. This would help determine whether the observed effects are consistent across different athletic populations and sports contexts, providing more generalizable insights into the role of BCAA supplementation in enhancing exercise performance.

## 5 Conclusion

The results indicated that BCAA supplementation can enhance RFD in basketball players, especially at the 50 ms interval. Our research design provides reliable insights into the effects of BCAAs on athletic performance. These findings highlight the potential for BCAA supplementation to improve rapid muscle response and explosive movement, which are essential for enhancing agility and performance in basketball. Further similar studies with larger sample sizes and additional biological and physical indicators are needed to validate and expand upon these findings.

## Data Availability

The original contributions presented in the study are included in the article/Supplementary material, further inquiries can be directed to the corresponding authors.

## References

[1] Tillin NA Jimenez-Reyes P Pain MT Folland JP. Neuromuscular performance of explosive power athletes versus untrained individuals. Med Sci Sports Exerc. (2010) 42:781–90. doi: 10.1249/MSS.0b013e3181be9c7e, PMID: 19952835

[2] Tillin NA Pain MTG Folland J. Explosive force production during isometric squats correlates with athletic performance in rugby union players. J Sports Sci. (2013) 31:66–76. doi: 10.1080/02640414.2012.720704, PMID: 22938509

[3] Aagaard P Simonsen EB Andersen JL Magnusson P Dyhre-Poulsen P. Increased rate of force development and neural drive of human skeletal muscle following resistance training. J Appl Physiol (1985)2002;93:1318–1326. doi: 10.1152/japplphysiol.00283.2002, PMID: 12235031

[4] Mangine GT Huet K Williamson C Bechke E Serafini P Bender D. A resisted Sprint improves rate of force development during a 20-m Sprint in athletes. J Strength Cond Res. (2018) 32:1531–7. doi: 10.1519/JSC.0000000000002030, PMID: 29786621

[5] Townsend JR Bender D Vantrease WC Hudy J Huet K Williamson C. Isometric Midthigh pull performance is associated with athletic performance and sprinting kinetics in division I men and Women's basketball players. J Strength Cond Res. (2019) 33:2665–73. doi: 10.1519/JSC.0000000000002165, PMID: 28777249

[6] Comfort P Allen M Graham-Smith P. Comparisons of peak ground reaction force and rate of force development during variations of the power clean. J Strength Cond Res. (2011) 25:1235–9. doi: 10.1519/JSC.0b013e3181d6dc0d, PMID: 21522071

[7] Gillen ZM Burch RF Saucier DN Strawderman L Luczak T Piroli A. Effects of a strength and conditioning offseason program on countermovement jump ground reaction forces in division I American football players. J Strength Cond Res. (2024) 38:e86–95. doi: 10.1519/JSC.0000000000004660, PMID: 38088878

[8] Haff GG Ruben RP Lider J Twine C Cormie P. A comparison of methods for determining the rate of force development during isometric midthigh clean pulls. J Strength Cond Res. (2015) 29:386–95. doi: 10.1519/JSC.0000000000000705, PMID: 25259470

[9] Comfort PS ThomasBeckham GK Stone MH Guppy SN Haff G. Gregory. Standardization and methodological considerations for the isometric Midthigh pull. Strength Cond J. (2019) 41:57–79. doi: 10.1519/SSC.0000000000000433

[10] Kuitunen S Komi PV Kyröläinen H. Knee and ankle joint stiffness in sprint running. Med Sci Sports Exerc. (2002) 34:166–73. doi: 10.1097/00005768-200201000-00025, PMID: 11782663

[11] Hernández-Davó JL Sabido R. Rate of force development: reliability, improvements and influence on performance. A review. Motricidad European. J Hum Mov. (2014) 1:28–42.

[12] Del Vecchio A Negro F Holobar A Casolo A Folland JP Felici F. You are as fast as your motor neurons: speed of recruitment and maximal discharge of motor neurons determine the maximal rate of force development in humans. J Physiol. (2019) 597:2445–56. doi: 10.1113/JP277396, PMID: 30768687 PMC6487919

[13] Andersen LL Andersen JL Zebis MK Aagaard P. Early and late rate of force development: differential adaptive responses to resistance training? Scand J Med Sci Sports. (2010) 20:e162–9. doi: 10.1111/j.1600-0838.2009.00933.x, PMID: 19793220

[14] Del Vecchio A. Neuromechanics of the rate of force development. Exerc Sport Sci Rev. (2023) 51:34–42. doi: 10.1249/JES.0000000000000306, PMID: 36123735

[15] Millet GY Lepers R. Alterations of neuromuscular function after prolonged running, cycling and skiing exercises. Sports Med. (2004) 34:105–16. doi: 10.2165/00007256-200434020-00004, PMID: 14965189

[16] Ament W Verkerke GJ. Exercise and fatigue. Sports Med. (2009) 39:389–422. doi: 10.2165/00007256-200939050-00005, PMID: 19402743

[17] Gandevia SC. Some central and peripheral factors affecting human motoneuronal output in neuromuscular fatigue. Sports Med. (1992) 13:93–8. doi: 10.2165/00007256-199213020-00004, PMID: 1561512

[18] Meeusen R Watson P Hasegawa H Roelands B Piacentini MF. Central fatigue: the serotonin hypothesis and beyond. Sports Med. (2006) 36:881–909. doi: 10.2165/00007256-200636100-00006, PMID: 17004850

[19] Petibois C Cazorla G Poortmans JR Déléris G. Biochemical aspects of overtraining in endurance sports: the metabolism alteration process syndrome. Sports Med. (2003) 33:83–94. doi: 10.2165/00007256-200333020-00001, PMID: 12617688

[20] Blomstrand E. A role for branched-chain amino acids in reducing central fatigue. J Nutr. (2006) 136:544s–7s. doi: 10.1093/jn/136.2.544S, PMID: 16424144

[21] Meeusen R Watson P. Amino acids and the brain: do they play a role in "central fatigue"? Int J Sport Nutr Exerc Metab. (2007) 17:S37–46. doi: 10.1123/ijsnem.17.s1.s37, PMID: 18577773

[22] Blomstrand E. Amino acids and central fatigue. Amino Acids. (2001) 20:25–34. doi: 10.1007/s007260170063, PMID: 11310928

[23] Martinho DV Nobari H Faria A Field A Duarte D Sarmento H. Oral branched-chain amino acids supplementation in athletes: a systematic review. Nutrients. (2022) 14:4002. doi: 10.3390/nu14194002, PMID: 36235655 PMC9571679

[24] Douligeris A Methenitis S Lazou A Panayiotou G Feidantsis K Voulgaridou G. The effect of acute pre-workout supplement ingestion on basketball-specific performance of well-trained athletes. Nutrients. (2023) 15:2304. doi: 10.3390/nu15102304, PMID: 37242187 PMC10220844

[25] Conte D Straigis E Clemente FM Gómez M Tessitore A. Performance profile and game-related statistics of FIBA 3x3 basketball world cup 2017. Biol Sport. (2019) 36:149–54. doi: 10.5114/biolsport.2019.83007, PMID: 31223192 PMC6561230

[26] Montgomery PG Maloney BD. Three-by-three basketball: inertial movement and physiological demands during elite games. Int J Sports Physiol Perform. (2018) 13:1169–74. doi: 10.1123/ijspp.2018-0031, PMID: 29584513

[27] Sansone P Conte D Tessitore A Rampinini E Ferioli D. A systematic review on the physical, physiological, perceptual, and technical-tactical demands of official 3 × 3 basketball games. Int J Sports Physiol Perform. (2023) 18:1233–45. doi: 10.1123/ijspp.2023-0104, PMID: 37567576

[28] Montgomery PG Maloney BD. 3×3 basketball: performance characteristics and changes during elite tournament competition. Int J Sports Physiol Perform. (2018) 13:1349–56. doi: 10.1123/ijspp.2018-0011, PMID: 29745788

[29] Khemtong C Tessitore A Jaime SJ Gobbi G Jensen J Yang AL. Branched-chain amino acids supplementation does not accelerate recovery after a change of direction sprinting exercise protocol. Nutrients. (2022) 14:4331. doi: 10.3390/nu14204331, PMID: 36297014 PMC9609908

[30] Cheng IS Wang YW Chen IF Hsu GS Hsueh CF Chang CK. The supplementation of branched-chain amino acids, arginine, and Citrulline improves endurance exercise performance in two consecutive days. J Sports Sci Med. (2016) 15:509–15. PMID: 27803630 PMC4974864

[31] Beckham GK Sato K Santana HAP Mizuguchi S Haff GG Stone MH. Effect of body position on force production during the isometric Midthigh pull. J Strength Cond Res. (2018) 32:48–56. doi: 10.1519/JSC.0000000000001968, PMID: 28486331

[32] Faul F Erdfelder E Lang AG Buchner A. G*power 3: a flexible statistical power analysis program for the social, behavioral, and biomedical sciences. Behav Res Methods. (2007) 39:175–91. doi: 10.3758/BF03193146, PMID: 17695343

[33] Cohen J. Statistical power analysis for the behavioral sciences. (2nd ed.). Routledge (1988).

[34] Hopkins WG Marshall SW Batterham AM Hanin J. Progressive statistics for studies in sports medicine and exercise science. Med Sci Sports Exerc. (2009) 41:3–12. doi: 10.1249/MSS.0b013e31818cb278, PMID: 19092709

[35] Rhea MR. Determining the magnitude of treatment effects in strength training research through the use of the effect size. J Strength Cond Res. (2004) 18:918–20. doi: 10.1519/00124278-200411000-00040, PMID: 15574101

[36] McMaster DT Gill N Cronin J McGuigan M. A brief review of strength and ballistic assessment methodologies in sport. Sports Med. (2014) 44:603–23. doi: 10.1007/s40279-014-0145-2, PMID: 24497158

[37] Dudgeon WD Kelley EP Scheett TP. In a single-blind, matched group design: branched-chain amino acid supplementation and resistance training maintains lean body mass during a caloric restricted diet. J Int Soc Sports Nutr. (2016) 13:1. doi: 10.1186/s12970-015-0112-9, PMID: 26733764 PMC4700774

[38] Areces F Salinero JJ Abian-Vicen J González-Millán C Gallo-Salazar C Ruiz-Vicente D. A 7-day oral supplementation with branched-chain amino acids was ineffective to prevent muscle damage during a marathon. Amino Acids. (2014) 46:1169–76. doi: 10.1007/s00726-014-1677-3, PMID: 24477835

[39] Stepto NK Shipperd BB Hyman G McInerney B Pyne DB. Effects of high-dose large neutral amino acid supplementation on exercise, motor skill, and mental performance in Australian rules football players. Appl Physiol Nutr Metab. (2011) 36:671–81. doi: 10.1139/h11-073, PMID: 21980992

[40] Spillane M Emerson C Willoughby DS. The effects of 8 weeks of heavy resistance training and branched-chain amino acid supplementation on body composition and muscle performance. Nutr Health. (2012) 21:263–73. doi: 10.1177/0260106013510999, PMID: 24620007

[41] Kephart WC Mumford PW McCloskey AE Holland AM Shake JJ Mobley CB. Post-exercise branched chain amino acid supplementation does not affect recovery markers following three consecutive high intensity resistance training bouts compared to carbohydrate supplementation. J Int Soc Sports Nutr. (2016) 13:30. doi: 10.1186/s12970-016-0142-y, PMID: 27468258 PMC4962429

[42] Newsholme EA Blomstrand E Ekblom B. Physical and mental fatigue: metabolic mechanisms and importance of plasma amino acids. Br Med Bull. (1992) 48:477–95. doi: 10.1093/oxfordjournals.bmb.a072558, PMID: 1360309

[43] Vigh-Larsen JF Ørtenblad N Spriet LL Overgaard K Mohr M. Muscle glycogen metabolism and high-intensity exercise performance: a narrative review. Sports Med. (2021) 51:1855–74. doi: 10.1007/s40279-021-01475-0 33900579

[44] Newsholme EA Blomstrand E. Branched-chain amino acids and central fatigue. J Nutr. (2006) 136:274s–6s. doi: 10.1093/jn/136.1.274S, PMID: 16365097

[45] Pardridge WM. Kinetics of competitive inhibition of neutral amino acid transport across the blood-brain barrier. J Neurochem. (1977) 28:103–8. doi: 10.1111/j.1471-4159.1977.tb07714.x, PMID: 833586

[46] Maffiuletti NA Aagaard P Blazevich AJ Folland J Tillin N Duchateau J. Rate of force development: physiological and methodological considerations. Eur J Appl Physiol. (2016) 116:1091–116. doi: 10.1007/s00421-016-3346-6, PMID: 26941023 PMC4875063

