# Supplementary material for: The effect of acute branched-chain amino acids ingestion on rate of force development in different time intervals: a controlled crossover study
Source: Front Nutr. 2025 Jan 8;11:1463202. doi: 10.3389/fnut.2024.1463202 (PMC11750653; doi:10.3389/fnut.2024.1463202)
Supplement: Supplementary file 1 [file Supplementary_file_1.docx]

The effect of acute branched-chain amino acids ingestion on rate of force development in different time intervals: A randomized, controlled crossover, double-blind trial

Xi-Nuan Zhang^1^, Long-Ji Li^2^, Yan-Hao Tu^2^, Li-Feng Zhang^2^, Hua-Yu Shang^3^, Meng Liu^4^, Ming-Da Li^1,*^

1 School of Sports Training, Chengdu Sport University, Chengdu City 610041, China

2 Strength and Conditioning Training Center, School of Physical Education, Chengdu Sport University, Chengdu City 610041, China

3 School of Sports Medicine and Health, Chengdu Sport University, Chengdu City 610041, China

4 Chongqing Institute of Sports Science, Chongqing City 400015, China

* Correspondence: [limingda@cdsu.edu.cn](mailto:limingda@cdsu.edu.cn)

**Figure S1.** Training supplements, field conditions, and testing. a: Packaging and coding of branched-chain amino acids (BCAAs) and placebo in the study. b−c: various field conditions observed during the experiment. d: RFD measurement.

**Figure S2.** flow chart of this study.

**Figure S3.** The change of peak force during the exercise protocol. (*) Denotes differences (p < 0.05) from all time-point measurements.

**Figure S4.** The change of vertical jump (VJ) fight time during the exercise protocol. (*) Denotes differences (p < 0.05) from all time-point measurements.

**Figure S5.** The change of vertical jump (VJ) height during the exercise protocol. (*) Denotes differences (p < 0.05) from all time-point measurements.

**Figure S6.** The change of reactive strength index (modify) test (RSImod) during the exercise protocol. (*) Denotes differences (p < 0.05) from all time-point measurements.

**Figure S7.** The change of peak power during the exercise protocol. (*) Denotes differences (p < 0.05) from all time-point measurements.

**Figure S8.** The change of mean power during the exercise protocol. (*) Denotes differences (p < 0.05) from all time-point measurements.

**Figure S9.** The change of 505 change of direction (COD) test during the exercise protocol. (*) Denotes differences (p < 0.05) from all time-point measurements.

**Figure S10.** The change of T test during the exercise protocol. (*) Denotes differences (p < 0.05) from all time-point measurements.

**Figure S11.** The change of blood lactate during the exercise protocol. (*) Denotes differences (p < 0.05) from all time-point measurements.

**Figure S12.** The change of the rating of perceived exertion (RPE) during the exercise protocol. (*) Denotes differences (p < 0.05) from all time-point measurements.


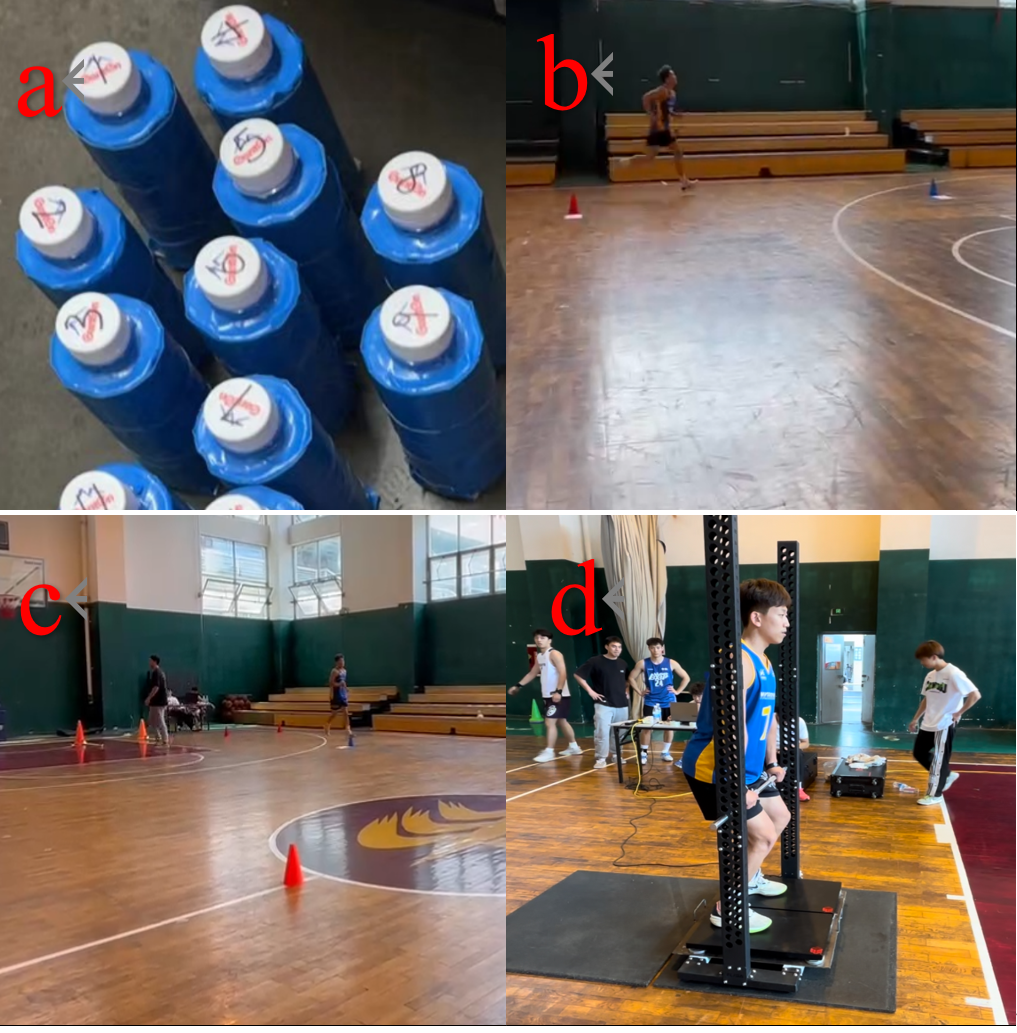


**Figure S1.** Training supplements, field conditions, and testing. a: Packaging and coding of branched-chain amino acids (BCAAs) and placebo in the study. b−c: various field conditions observed during the experiment. d: RFD measurement.

**
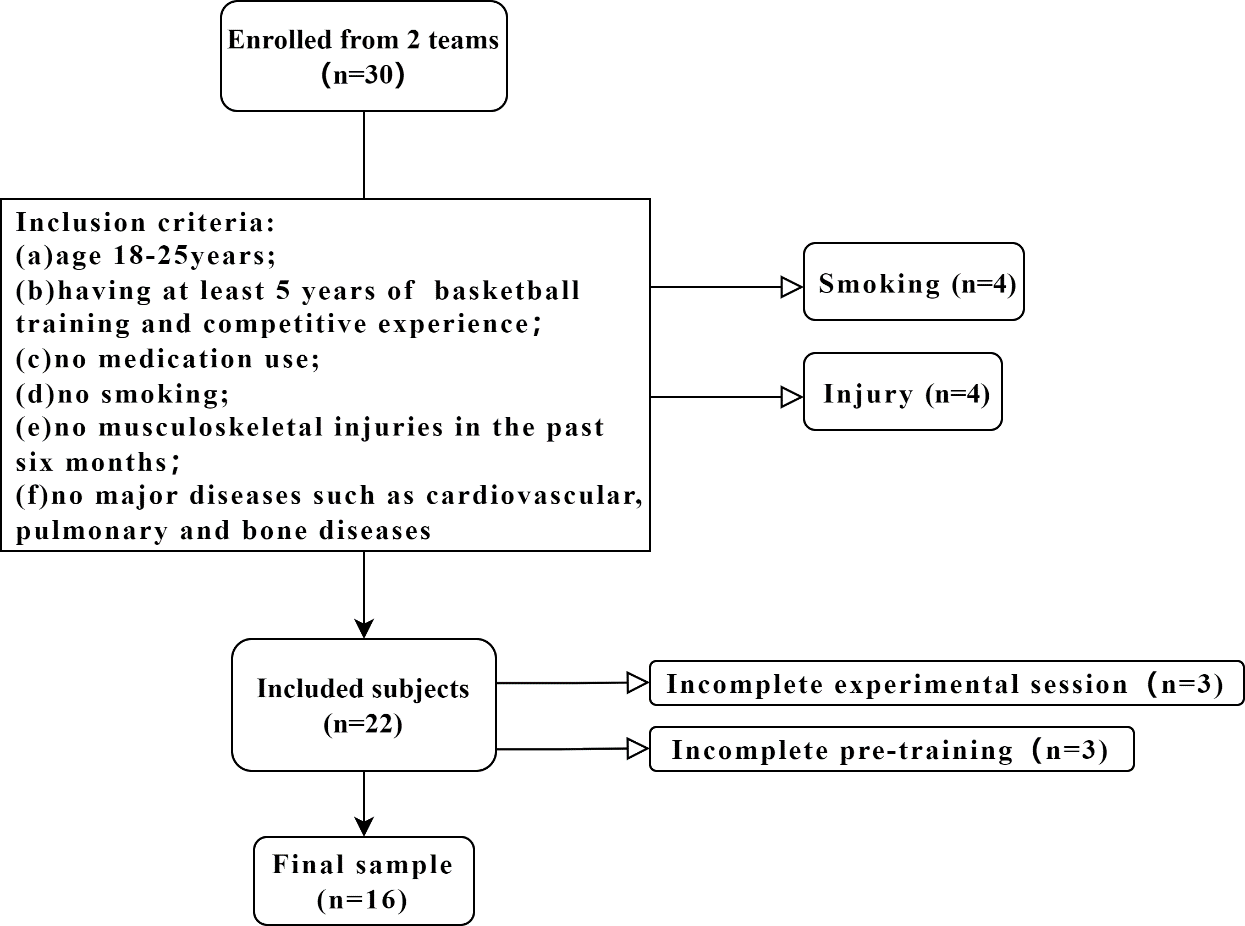
**

**Figure S2.** flow chart of this study.

**Figure S3.** The change of peak force during the exercise protocol. (*) Denotes differences (p < 0.05) from all time-point measurements.

 **Figure S4.** The change of vertical jump (VJ) fight time during the exercise protocol. (*) Denotes differences (p < 0.05) from all time-point measurements.

 **Figure S5.** The change of vertical jump (VJ) height during the exercise protocol. (*) Denotes differences (p < 0.05) from all time-point measurements.

 **Figure S6.** The change of reactive strength index （modify）test (RSImod) during the exercise protocol. (*) Denotes differences (p < 0.05) from all time-point measurements.

 **Figure S7.** The change of peak power during the exercise protocol. (*) Denotes differences (p < 0.05) from all time-point measurements.

 **Figure S8.** The change of mean power during the exercise protocol. (*) Denotes differences (p < 0.05) from all time-point measurements.

**Figure S9.** The change of 505 change of direction (COD) test during the exercise protocol. (*) Denotes differences (p < 0.05) from all time-point measurements.

**Figure S10.** The change of T test during the exercise protocol. (*) Denotes differences (p < 0.05) from all time-point measurements.

**Figure S11.** The change of blood lactate during the exercise protocol. (*) Denotes differences (p < 0.05) from all time-point measurements.

**Figure S12.** The change of the rating of perceived exertion (RPE) during the exercise protocol. (*) Denotes differences (p < 0.05) from all time-point measurements.
